# ﻿Three new *Pseudogymnoascus* species (*Pseudeurotiaceae*, *Thelebolales*) described from Antarctic soils

**DOI:** 10.3897/imafungus.16.e142219

**Published:** 2025-03-21

**Authors:** Mary K. Childress, Nicholas B. Dragone, Benjamin D. Young, Byron J. Adams, Noah Fierer, C. Alisha Quandt

**Affiliations:** 1 Department of Ecology and Evolutionary Biology, University of Colorado Boulder, Boulder, CO, USA; 2 Cooperative Institute for Research in Environmental Sciences, University of Colorado Boulder, Boulder, CO, USA; 3 Department of Biology, Evolutionary Ecology Laboratories, and Monte L. Bean Museum, Brigham Young University, Provo, UT, USA

**Keywords:** Antarctic microbial diversity, new species, phylogenetics, psychrophilic fungi, psychrotolerance, taxonomy, whole genome assembly

## Abstract

The genus *Pseudogymnoascus* includes several species frequently isolated from extreme environments worldwide, including cold environments such as Antarctica. This study describes three new species of *Pseudogymnoascus*—*P.russus***sp. nov.**, *P.irelandiae***sp. nov.**, and *P.ramosus***sp. nov.**—isolated from Antarctic soils. These species represent the first *Pseudogymnoascus* taxa to be formally described from Antarctic soil samples, expanding our understanding of fungal biodiversity in this extreme environment. Microscopic descriptions of asexual structures from living cultures, along with measurements of cultural characteristics and growth on various media types at different temperatures, identify three distinct new species. In addition, phylogenetic analyses based on five gene regions (ITS, LSU, MCM7, RPB2, TEF1) and whole-genome proteomes place these new species within three distinct previously described clades: *P.irelandiae* in clade K, *P.ramosus* in clade Q, and *P.russus* in clade B. These results provide further evidence of the extensive undescribed diversity of *Pseudogymnoascus* in high-latitude soils. This study contributes to the growing body of knowledge on Antarctic mycology and the broader ecology of psychrophilic and psychrotolerant fungi.

## ﻿Introduction

The genus *Pseudogymnoascus* (*Pseudeurotiaceae*, *Thelebolales*, *Leotiomycetes*) was first established in 1929 with the type *P.roseus* isolated from Russian soil (Raillo 1929; Samson 1972), followed by the description of *P.caucasicus*, *P.bhatti*, and *P.alpinus* from alpine soils also in the 1900s (Cejp and Milko 1966; Samson 1972; Müller and von Arx 1982). In the 21^st^ century, cold adapted *P.appendiculatus* and *P.verrucosus* were the first species described using a combination of ITS phylogenetic and morphological analyses (Rice and Currah 2006). The emergence and discovery of white-nose syndrome, a devastating disease of North American bats caused by *P.destructans* (=*Geomycesdestructans*), led to increased interest and research in this genus (Gargas et al. 2009; Minnis and Lindner 2013). Previous research has led to the re-assignment of several species from *Geomyces* and *Sporotrichum* to *Pseudogymnoascus* based on the first multigene (ITS, LSU, MCM7, RPB2, TEF1) phylogeny of this clade (Minnis and Lindner 2013) and the description of several new species from bat hibernacula in the USA (*P.lindneri*, *P.palmeri*, *P.turneri*) and Europe (*P.cavicola*) (Crous et al. 2019; Crous et al. 2020; Becker et al. 2023). Outside of North America and Europe, new species of *Pseudogymnoascus* have been described from a variety of environments. Twelve species have been described from Chinese soils (Zhang et al. 2020, 2021, 2023a, 2023b), and four species have been described from Antarctic marine sponges (Villanueva et al. 2021). Following the work of Minnis and Lindner (2013), new species of *Pseudogymnoascus* are described based on a combination of morphological characters and five gene phylogenetic analyses with an organization around clades (A-P) from isolates or environmental sequences (Villanueva et al. 2021; Zhang et al. 2023b). This system has helped researchers keep track of the existing, but mostly unnamed, diversity within this understudied genus.

As a genus, *Pseudogymnoascus* is known for thriving in extreme environments, with many taxa that are known to be cold-adapted. In Antarctica, *Pseudogymnoascus* is commonly isolated from soils, lakes, mosses, plants, macroalgae, lichens, and marine sponges despite the challenging environmental conditions often typical of these systems (Fell et al. 2006; Rosa 2019). Antarctic soils are generally colder and drier with higher salt concentrations and lower organic matter concentrations than soils in more temperate regions (Bockheim 1997). The ecological role of *Pseudogymnoascus* species across Antarctica remains unknown, but researchers speculate that they may contribute to decomposition and nutrient cycling (Arenz et al. 2006). We do not yet know if *Pseudogymnoascus* species found in Antarctica are dormant or metabolically active, however *in vitro* experiments have proven the ability of this genus to tolerate a range of challenging environmental conditions. For instance, *P.pannorums.l.*, cultured from cryopegs in Russia, was able to grow on media with high NaCl concentrations, at temperatures as low as -2°C, and produce spores after 2–3 weeks of growth (Kochkina et al. 2007). *Pseudogymnoascuspannorums.l.* cultured from Arctic soil was able to grow on silica gel with no added organic compounds (Bergero et al. 1999). An Antarctic strain of *P.pannorums.l.* was also shown to synthesize increased amounts of linoleic acid when grown at 8°C opposed to 25°C (Maggi et al. 2013). The production of unsaturated fatty acids is a common adaptation of psychrophilic fungi to increase fluidity of the cytoplasm, acting as an antifreeze within the cell membrane (Maggi et al. 2013).

While *P.pannorums.l.*, *P.destructans*, *P.appendiculatus*, and *P.verrucosus* have all been reported in Antarctica, several studies have reported unidentified *Pseudogymnoascus* taxa only identified to the genus level (Rosa 2019). In 2021, the first four *Pseudogymnoascus* species described from Antarctica were cultured from marine sponges: *P.antarcticus*, *P.australis*, *P.griseus*, and *P.lanuginosus* (Villanueva et al. 2021). This paper describes three additional *Pseudogymnoascus* species from Antarctica, representing the first members of *Pseudogymnoascus* described from Antarctic soil. This is also the first study to describe species in this genus with both phylogenetic and phylogenomic support, for which the names *P.irelandiae* sp. nov., *P.ramosus* sp. nov., and *P.russus* sp. nov. are proposed.

## ﻿Materials and methods

### ﻿Sampling and fungal isolation

Proposed novel *Pseudogymnoascus* species analyzed in this study were isolated from surface soil samples collected and archived from previous expeditions in 2004 and 2018 to Shackleton Glacier and Cape Hallett regions. Refer to Suppl. material 1: table S1 as well as Dragone et al. (2021) for additional sample collection details from the Antarctic expeditions. The holotypes were deposited in the Center for Forest Mycology Research (CFMR) fungarium, maintained by the USDA Forest Service, Northern Research Station and housed in the Forest Products Laboratory; Madison, WI, USA, as dried, metabolically inactive, specimens. Ex-type material, metabolically active specimens, were deposited in the culture collection of the CFMR. The majority of soil samples analyzed were a part of a previous study (Dragone et al. 2021), with the exception of samples 99ASP01 and 273ASP01. These samples were initially cultured by homogenizing 1 g of each soil sample with 1 mL of filtered Phosphate Buffered Saline 10X Solution and serially diluted (1/10, 1/100, and 1/1,000). 50 µL of each serial dilution were plated onto Potato Dextrose Agar (PDA; Difco Laboratories, Detroit, MI, USA) and spread across the plates using a flame sterilized inoculation loop. ‘Blank’ plates inoculated with 50 µL sterile water were prepared on PDA and handled in an identical manner to the plates inoculated with the soil slurries. All plates were incubated at 5°C in a VWR Laboratory Refrigerator in the dark. All plates (inoculated and blank) remained under these conditions for three months. Plates were checked weekly throughout the incubation and all fungal colonies that grew were isolated and transferred to new PDA plates.

### ﻿DNA extraction, amplification and sanger sequencing

DNA was extracted from all fungal culture isolates using the Qiagen DNeasy^®^ Plant Mini Kit (Qiagen, Germantown, MD, USA) following the manufacturer’s protocol. To identify cultures that were members of the genus *Pseudogymnoascus*, the internal transcribed spacer (ITS) region of all fungal isolates was sequenced (primer and PCR cycle details in Suppl. material 1: tables S2, S3). Sequence results were used in a BLASTn search in NCBI, and all fungal isolates with top blast results in the genus *Pseudogymnoascus* were selected for further phylogenetic analyses (see section below for more details). We amplified and sequenced four gene targets from the *Pseudogymnoascus* species: ITS, nuclear large subunit (LSU) ribosomal DNA (rDNA), DNA replication licensing factor (MCM7), and RNA polymerase II second largest subunit (RPB2) to identify unique species (Suppl. material 1: tables S2, S3). Translation elongation factor EF-1a (TEF1) was not able to be amplified successfully. Sanger sequencing of all amplicons was done by Genewiz (Azenta Life Sciences, Burlington, MA, USA).

### ﻿Genome sequencing, assembly, and annotation

Previously extracted genomic fungal DNA was shipped to Novogene for library preparation and Illumina 2x 150 Whole Genome Sequencing (WGS) for one strain of each of the three putative new *Pseudogymnoascus* species. Adapters and low-quality sequences from raw reads were removed using Trim Galore v0.6.10 (Krueger 2024) with default settings. Prior to genome assembly, *k*-mer profiles were generated using the trimmed raw reads and Meryl v1.3 (Rhie et al. 2020) and used for genome profiling in GenomeScope2 v2.0 (Ranallo-Benavidez et al. 2020). Trimmed raw reads were assembled using SPAdes v3.15.5 (Prjibelski et al. 2020) and assembly statistics were obtained using BUSCO v5.7.1, with the ascomycota_odb10 database (Manni et al. 2021), and Quast v5.2.0 (Mikheenko et al. 2016; Mikheenko et al. 2018). *De novo* libraries of repetitive elements were identified using RepeatModeler2 v2.0.5 (Flynn et al. 2020) before being passed to RepeatMasker v4.1.5 (Hubley 2024) to generate hard masked and soft masked versions of genome assemblies. Gene models were predicted using Funannotate::predict v1.8.15 (Palmer and Stajich 2019) using the soft masked genomes. Predicted gene models were then run through Interproscan v5.59-91.0 (Jones et al. 2014) to classify protein families and predict domains. Interproscan (Jones et al. 2014) and Funannotate::predict results were then used in Funannotate::annotate (Palmer and Stajich 2019) to annotate predicted protein-coding genes. The additional eggNOG-mapper v2.1.12 (Cantalapiedra et al. 2021) option was also incorporated.

### ﻿Phylogenetic and phylogenomic analyses

First, phylogenetic analysis of ITS from all *Pseudogymnoascus* strains obtained in this study were aligned using MUSCLE v.5.1 (Edgar 2021) with default parameters. Aligned sequences were manually trimmed in BioEdit v.7.7 removing bases present in less than 75% of the taxa. Maximum likelihood analyses were performed with RAxML-HPC2 v8.2.12 (Stamatakis 2014) on ACCESS (Towns et al. 2014) with a Lewis standard correction for ascertainment bias correction (Lewis 2001). GTRGAMMA RAxML v7.2.0 was used in the bootstrapping phase with bootstrap analyses performed using 100 replicates. Unique *Pseudogymnoascus* genotypes from the three clades identified via the ITS phylogenetic analysis were then used in the phylogenetic analysis of four genes (ITS, LSU, MCM7, RPB2), and included other publicly available *Pseudogymnoascus* species and strains (Suppl. material 1: table S4); For genotypes with multiple isolates, two morphologically similar isolates were randomly selected for further analyses. All gene sequences were aligned and trimmed following the same steps as the ITS phylogenetic analysis. The aligned and trimmed gene sequences were concatenated in MEGA v11 (Tamura et al. 2021). Maximum likelihood analysis following the same steps as the ITS phylogenetic analysis but with the concatenated sequence data. Proposed novel *Pseudogymnoascus* species that were identified from the four-gene phylogenetic analysis were then used in downstream morphological analysis and whole-genome sequencing.

To build a five-gene phylogeny, ITS, LSU, MCM7, RPB2, and TEF1 were manually extracted from the assembled genomes using BLAST Command Line Applications v2.15.0+ (Madden and Camacho 2021) with Genbank deposited sequences for *P.destructans*, and aligned, trimmed, concatenated, and phylogenetically analyzed using the same protocol as described above (Suppl. material 1: tables S2, S3, S4). Sequences of newly described species have been deposited in the Genbank database with the accession numbers listed in Suppl. material 1: table S4 and the Data Availability section.

A phylogenomic analysis was run using protein sequences from the genome assemblies as well as a number of additional *Pseudogymnoascus* proteomes (Suppl. material 1: table S5). Protein sequences were run in Proteinortho6 (Lechner et al. 2011) to identify orthologous clusters across the protein files. Single-copy orthologous clusters were then extracted and aligned using MUSCLE v.5.1 (Edgar 2021) with default parameters. TrimAL v2.rev0 (Capella-Gutiérrez et al. 2009) was run on the muscle output to trim the alignment. Sequences were then concatenated, using Bioperl v1(Stajich et al. 2002), and RAxML (Stamatakis 2014) was subsequently run on the concatenated sequence data under the PROTGAMMAAUTO model of amino acid substitution with support from 100 bootstrap replicates. Other publicly available *Thelebolales* taxa, *Pseudeurotiumzonatum* (NCBI PRJNA1181865) and *Antarctomycespellizariae* (Batista et al. 2020), were used as outgroup taxa.

### ﻿Morphological analyses

All proposed novel *Pseudogymnoascus* isolates were plated on PDA, Sabouraud Dextrose Agar (SDA; Difco Laboratories, Detroit, MI, USA), Corn Meal Agar (CMA; Difco Laboratories, Detroit, MI, USA), and Oatmeal Agar (OA; 30 g oatmeal, 1 L water, 15 g agar), and incubated at 5°C in a VWR Laboratory Refrigerator in the dark, as well as at 15°C in a Thermo Scientific MaxQ 6,000 Incubator Shaker in the dark. All media preparations, bar OA, followed manufacturer’s protocols. Fresh subcultures from spore glycerol stocks stored at -80°C were used for all colony characteristic observations, documented after 28 days of growth on the four media types; the presence of soluble pigments and/or exudates, the obverse and reverse colors of colonies, color of mycelia, and colony diameters were observed. Additionally, to allow direct comparisons with other descriptions of new *Pseudogymnoascus* species characterized at higher temperatures, (Zhang et al. 2020, 2021, 2023a, 2023b) and to see how time and temperature affects colony morphology, we documented colony morphology observed on PDA at 14 and 28 days at 15°C and 25°C with plates incubated in shaker incubators (ThermoScientific MaxQ 6,000). The Methuen Handbook of Colour (Kornerup and Wanscher 1978) was used as the color guide for the description of colony morphology.

All light microscopy images were taken using an Olympus BX43 microscope and Olympus SC50 camera with the OLYMPUS cellSens standard software. Microscopic characteristics, from CMA and PDA cultures, were examined and measured after 7 and 14 days of growth at 15°C and mounted in 25% lactic acid (Fisher Scientific, Waltham, MA, USA). The presence of sexual structures was checked on OA at 14 days and at 2, 4, 6, and 8 months of cultivation.

## ﻿Results

### ﻿Isolation of *Pseudogymnoascus* from Antarctic soils

Plated Antarctic soils yielded a total of 66 isolates of *Pseudogymnoascus* grown on PDA. No colonies were observed on any of the uninoculated control plates. While ITS phylogenies are generally not powerful enough to separate different *Pseudogymnoascus* taxa (Suppl. material 2), we were able to identify three unique genotypes based on the initial ITS phylogenetic analysis (Suppl. material 3) from the Antarctic soils; 55 grouping with *P.russus* sp. nov., 10 grouping with *P.ramosus* sp. nov., and one with *P.irelandiae* sp. nov. We selected isolates 99ASP01 and 390ASP from the first genotype, 420ASP and 508ASP from the second genotype, and 273ASP01 from the third genotype to be used in downstream analyses. Four gene phylogenetic analysis, with the addition of previously studied *Pseudogymnoascus* species and strains (Suppl. material 4), confirmed that these isolates from three unique genotypes represent three new species-level lineages, and those taxa are described here.

### ﻿Phylogenetic and phylogenomic analyses

**Figure 1.**
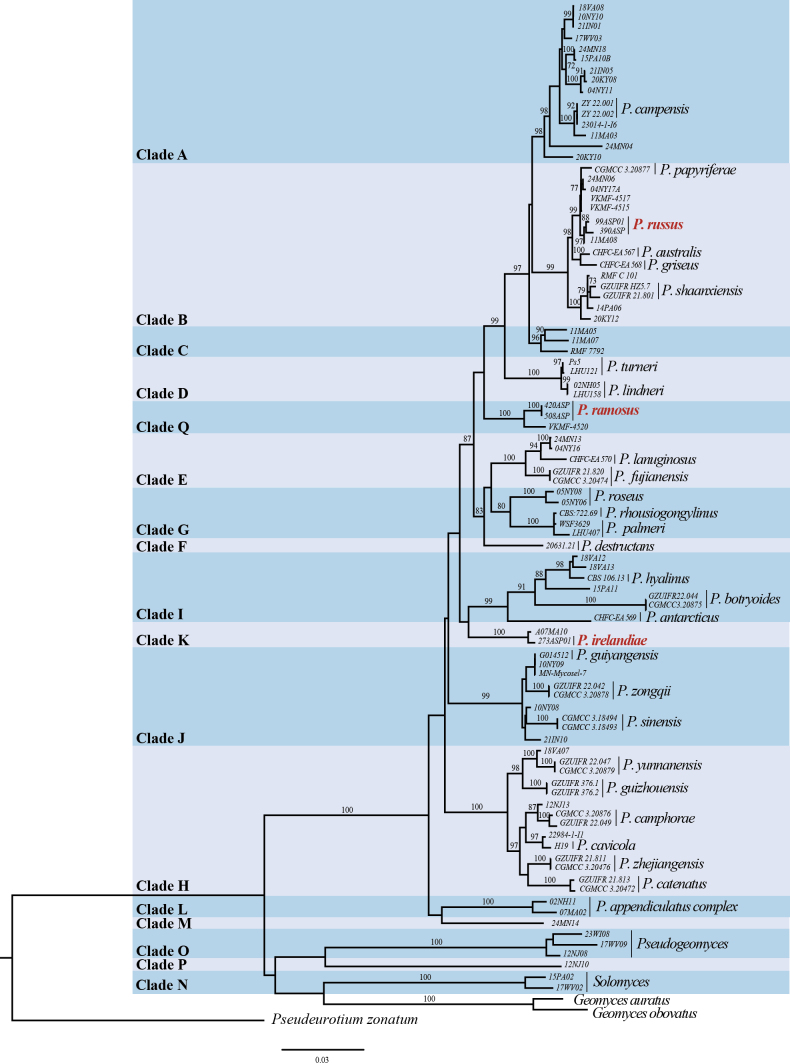
Maximum likelihood (ML) phylogenetic tree of *Pseudogymnoascus* based on the concatenated dataset of five genes (ITS, LSU, MCM7, RPB2, TEF1). ML bootstrap values ≥ 70 are shown above branches. New species described here are highlighted in bold and red. The scale bar indicates 0.03 nucleotide changes per site.

To refine our phylogenetic analyses, we sequenced the genomes of one strain of each of the three putative new species. Genome assemblies ranged in size from 32.4–33.4 Mb, had BUSCO completion between 96.1% and 97.1%, and had between 11,037 and 11,530 predicted genes. Full genome statistics and results are available in Suppl. material 1: table S7. Genes extracted from genomes were 100% similar to those from Sanger sequencing. The five-gene phylogeny (Fig. 1) had a concatenated alignment containing 3,974 nucleotides (ITS: 513; LSU: 1,198; MCM7: 598; RPB2: 761; TEF1: 904). The Maximum Likelihood tree (Fig. 1) shows the three newly described species placed on well supported branches in clades B, K, and Q. Previously described species of Antarctic origin are in clades B (*P.australis* and *P.griseus*), E (*P.lanuginosus*), and I (*P.antarcticus*). New to this study, *P.ramosus* sp. nov. is the first species to be described within clade Q, sister to the arctic strain VKM F-4520 which was isolated from the active layer of permafrost in the Kolyma lowland of Yakutia, Russia (Leushkin et al. 2015). And *P.irelandiae* is the first described species in clade K, sister to strain A07MA10 that was isolated from hibernacula soil from Massachusetts, USA (Minnis and Lindner 2013). Within the more well studied clade B, *P.russus* is sister to *P.papyriferae*, which was isolated from soil in Hanzhong City, China, in addition to strains 11MA08, 04NY17A, and 24MN06 isolated from hibernacula soils in the USA (Minnis and Lindner 2013; Zhang et al. 2023b).

**Figure 2.**
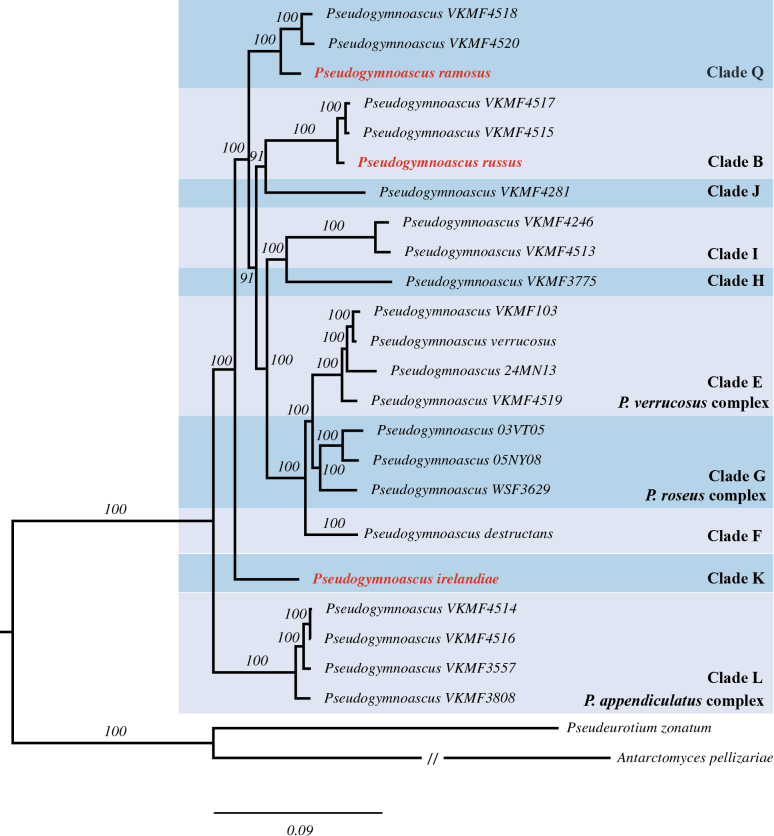
ML phylogeny of *Pseudogymnoascus* based on an alignment of 1496 concatenated orthogroups with single-copy genes. ML bootstrap values ≥ 75 are shown above branches. New species are highlighted in bold and red. The scale bar indicates 0.09 amino acid changes per site.

The genome-scale protein cluster phylogeny (Fig. 2) reflects many of the same clades that have been previously established in multi gene trees (Minnis and Lindner 2013) with our results reaffirming clade support for B, E, F, H, I, J, L, and Q that has previously been shown in a previously published *Pseudogymnoascus* genome-scale phylogeny (Reynolds et al. 2016). Fig. 2 also supports clade G that has been well documented in four to five gene trees. The addition of *P.irelandiae* appears to add greater support to clade K which has continuously had low basal boot strap and posterior probability support in *Pseudogymnoascus* five gene trees (Minnis and Lindner 2013; Zhang et al. 2023b). The protein tree generated in this study, in addition to the five gene tree, show strong support for the placement of *P.russus* in clade B, *P.ramosus* in clade Q, and *P.irelandiae* in clade K. However, the relationship and placement between clades within the genus in genome-scale trees (Fig. 2), (Reynolds et al. 2016) differ from that of five gene trees (Fig. 1), (Villanueva et al. 2021; Zhang et al. 2021, 2023). An exception to this is the relationship between clades E, F, and G, which are recovered in both our five gene and genome-scale trees as clade F being sister to clades E and G (Figs 1, 2). Despite some deeper nodes being poorly supported and unresolved, the nodes of the new taxa described here are well-supported.

### ﻿Taxonomy

Phylogenetics at both the gene and genome scale show strong support for *P.irelandiae*, and *P.ramosus*, and *P.russus* being characterized as new species. Differences in culture characteristics and microscopic features between these newly described species and previously described ones further support the formal addition of these three species to the genus *Pseudogymnoascus*. See Suppl. material 1: table S6 for a comparison of culture characteristics and microscopic features of Antarctic species.

#### ﻿ Pseudogymnoascus irelandiae

Taxon classificationAnimaliaThelebolalesPseudeurotiaceae

Childress & Quandt sp. nov

0C59BD40-9B78-5EF5-B4C7-943FF5A2B90E

856261

Figs 3 , 4

##### Etymology.

Named after Abigail Ireland for her substantial contributions to the taxonomy of *Pseudogymnoascus*.

##### Type.

Antarctica • Cape Hallett, 72°19'16.57"S, 170°13'41.58"E, 2 m, from soil, 14 Dec 2004, coll. B. Adams. Holotype 273ASP01, stored in a metabolically inactive state in the CFMR Herbarium, while ex-type metabolically active material is stored in the Reference Culture Collection at the CFMR.

**Figure 3.**
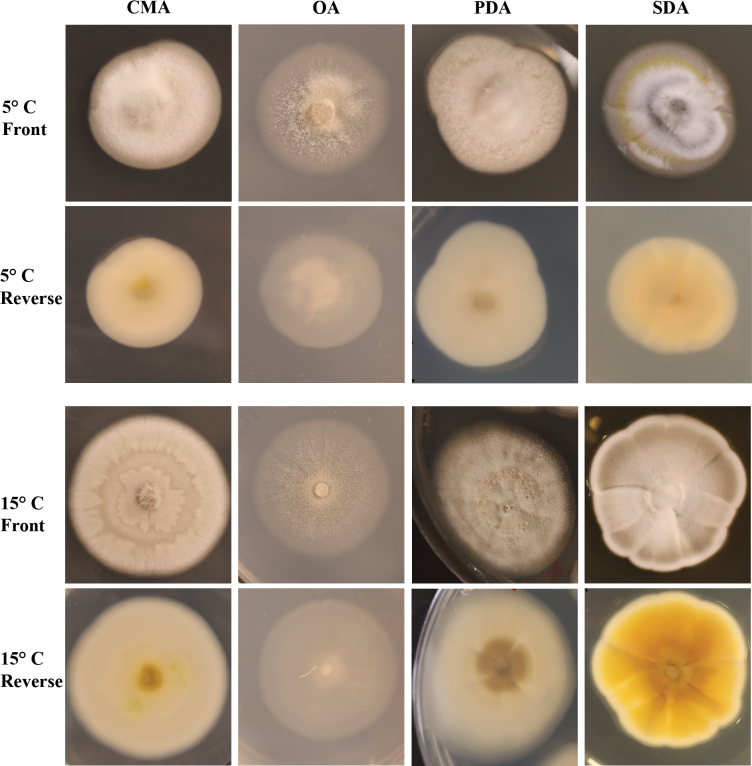
*Pseudogymnoascusirelandiae* sp. nov. colony morphology at 5°C and 15°C after 28 days on CMA, OA, PDA, and SDA.

##### Description.

On CMA and PDA hyphae branched, septate, hyaline, smooth, 0.9–1.9 μm wide. Coiled hyphae sometimes found on CMA. Hyphae form tight bundles of 3–11 hyphae on PDA. Racquet hyphae absent. Fertile hyphae bearing aleurioconidia, sessile or stalked. Arthroconidia not observed. Conidiophores abundant, solitary, usually curved, occasionally erect, arising in acute angles with the main axis, hyaline, smooth, usually bearing verticils of two to four branches arising from the stipe at an acute angle. Conidiophores more abundant on CMA than PDA. Aleurioconidia are pyriform to clavate or obovoid with a broad truncate basal scar, 2.8–4.6 × 1.7–3.2 μm (av = 3.7 × 2.5 μm, n = 50), in conidiophores separated by connective cells. Intercalary conidia are rare, pyriform to clavate, or subglobose, 2.4–3.9 × 1.6–2.4 μm (av = 3.1 × 2.0 μm, n = 11), in conidiophores separated by connective cells. Ascomata absent.

**Figure 4.**
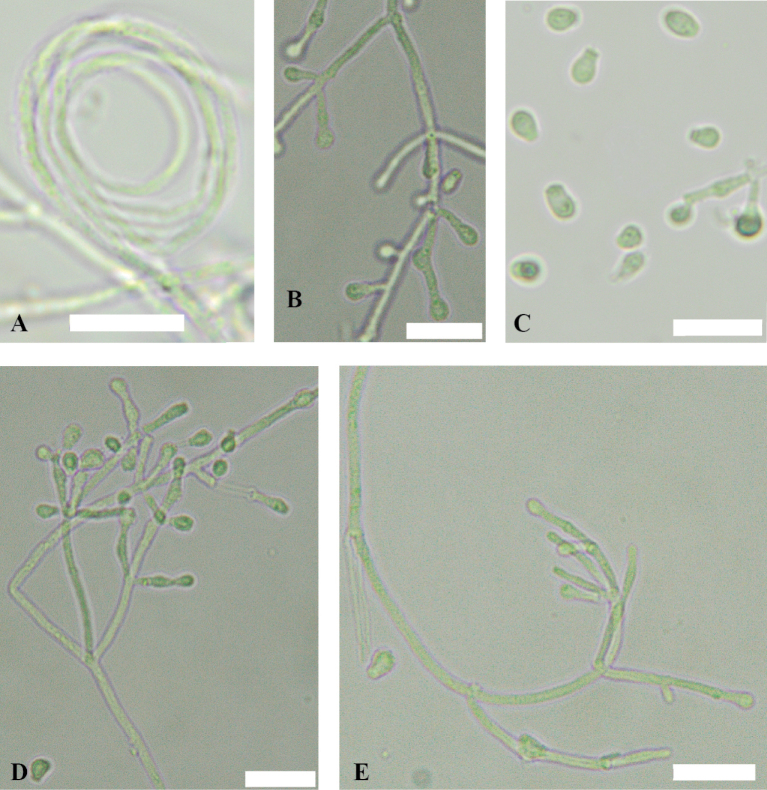
*Pseudogymnoascusirelandiae* sp. nov. **A** coiled hyphae **B** fertile hyphae bearing aleurioconidia **C** conidia **D, E** conidiophores. Scale bars: 10 µm.

##### Culture characteristics.

On OA, colonies reach 44 mm in diameter after 28 days at 15°C, round, appressed, colorless to white, consisting of immersed and hyaline hyphae, small spots of white cottony aerial mycelium emerging throughout the colony, exudates and diffusible pigments absent; reverse white. On CMA, colonies reach 36 mm in diameter after 28 days at 15°C, round, flat, floccose, gray to white, forming irregular concentric rings, filamentous margin, exudates and diffusible pigments absent; reverse white, brown to yellow at center. On SDA, colonies reach 43 mm in diameter after 28 days at 15°C, irregular, slightly raised, floccose, shallow radial grooves, white to gray, margin filamentous and white, sparse exudates in the form of small transparent and colorless droplets, diffusible pigments absent; reverse light brown to yellow. On PDA, colonies reach 35 mm in diameter after 28 days at 15°C, round, slightly raised, floccose, white, white cottony aerial mycelium emerging throughout the colony, exudates in the form of transparent and colorless droplets, diffusible pigments absent; reverse tan to cream. Growth occurred at 5°C and 15°C, with very minimal growth at 25°C; Optimum growth was observed at 15°C. No culture attenuation was observed.

##### Distribution.

Cape Hallett, Antarctica.

##### Ecology/substrate.

Cultured from Antarctic soil.

##### Genbank accession numbers.

ITS = PQ453553, LSU = PQ453558, MCM7 = PQ497089, RPB2 = PQ497096, TEF1 = PQ497101

##### NCBI BioSample genome accession.

SAMN40283453.

##### Note.

*Pseudogymnoascusirelandiae* has been placed as a member of clade K (Figs 1, 2), which also includes unidentified strain A07MA10 (Minnis and Lindner 2013). Both *Pseudogymnoascusirelandiae* and strain A07MA10 have a TEF1 amino acid insert at the same position that no other *Pseudogymnoascus* species have (Minnis and Lindner 2013). Fig. 1 shows clade K as sister to clade I, however the placement of clade K in relation to other clades has low bootstrap support. The placement of this clade has continued to have low support since it was originally classified in Minnis and Lindner (2013). However, according to Fig. 2, there is strong bootstrap support for clade K being sister to clades B, E, F, G, H, I, J, Q; but data are missing for clades A, C, and D to confirm this placement. *Pseudogymnoascusirelandiae* can be distinguished from species in clade B by its presence of coiled hyphae. And differentiated from *P.lanuginosus* and *P.fujianensis* in clade E by smaller aleurioconidia size (Villanueva et al. 2021; Zhang et al. 2021). Within clade F, *P.destructans* (Minnis and Lindner 2013) has asymmetrically curved conidia not observed in *P.irelandiae*. Clade G consists of *P.palmeri*, *P.roseus*, and *P.rhousiogongylinus* (Wener and Cain 1970; Crous et al. 2019, 2020), all of which have ascomata while *P.irelandiae* does not. *Pseudogymnoascusirelandiae* differs from *P.yunnanensis*, *P.guizhouensis*, *P.camphorae*, *P.cavicola*, *P.zhejianensis*, and *P.catensis* in clade H based on its presence of coiled hyphae (Zhang et al. 2020, 2021, 2023b; Becker et al. 2023). *Pseudogymnoascusirelandiae* differs from *P.hyalinus* by its lack of coremia (Daszewska 1924), and from *P.botryoides* and *P.antarcticus* in clade I with its lack of arthroconidia (Villanueva et al. 2021; Zhang et al. 2023b). Within clade J are *P.guiyangensis*, *P.zongqii*, and *P.sinensis*, in addition to undescribed strains 10NY09, MN-Mycosel-7, 10NY08, and 21IN10 (Minnis and Lindner 2013; Luo et al. 2016; Zhang et al. 2020, 2023b). *Pseudogymnoascusirelandiae* has a smaller hyphal width than *P.guiyangensis* (0.9–1.9 μm vs 1.5–2.5 μm). *Pseudogymnoascuszongqii* lacks exudates on PDA whereas *P.irelandiae* has colorless exudates. *Pseudogymnoascusirelandiae* has white colonies on PDA whereas *P.sinensis* has a light pink center. Lastly, *P.irelandiae* differs from *P.ramosus* in clade Q in its larger colony diameter on PDA, OA, SDA, and CMA, lack of gregariously branching groups of conidiophores, and different exudate colors. Phylogenetically, *P.irelandiae* forms a well-supported independent lineage with strain A07MA10, with a bootstrap value of 100, being the first described species in clade K. We are not able to confirm if strain A07MA10 is conspecific with *P.irelandiae* as we don’t have access to this isolate to analyze its culture and microscopic characteristics; however, we do know that their isolation sources are quite different, as A07MA10 was cultured from soil from bat hibernacula in Massachusetts, USA (Minnis and Lindner 2013).

#### ﻿ Pseudogymnoascus ramosus

Taxon classificationAnimaliaThelebolalesPseudeurotiaceae

Childress & Quandt sp. nov

4A4D489E-246A-5143-91BE-E134D56E0B8F

856262

Figs 5 , 6

##### Etymology.

The name refers to heavily branched hyphae with gregarious groupings of conidiophores.

##### Type.

Antarctica • Shackleton Glacier, Mount Franke, 84°37'35.52"S, 176°44'36.12"W, 485 m, from soil, 2 Jan 2018, coll. B. Adams, G. Schellens, N. Fierer & M. Shaver-Adams. Holotype 420ASP, stored in a metabolically inactive state in the CFMR Herbarium, while ex-type metabolically active material is stored in the Reference Culture Collection at the CFMR.

**Figure 5.**
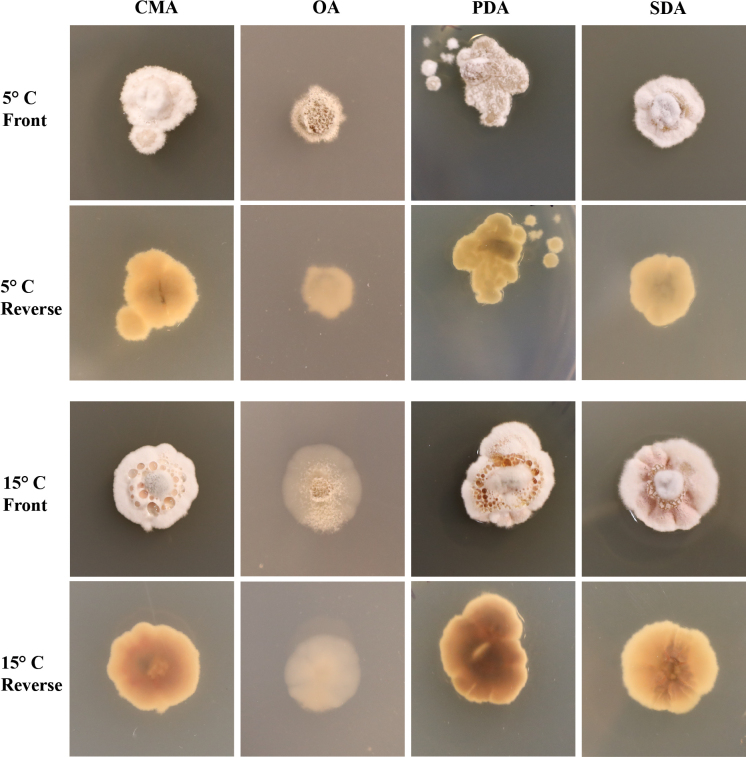
*Pseudogymnoascusramosus* sp. nov. colony morphology at 5°C and 15°C after 28 days on CMA, OA, PDA, and SDA.

##### Description.

On CMA and PDA hyphae branched, septate, hyaline, smooth, 0.9–1.9 μm wide. Racquet hyphae absent. Fertile hyphae bearing aleurioconidia, sessile or stalked. Arthroconidia not observed. Conidiophores abundant, often grouping gregariously but sometimes solitary, erect, arising in acute angles with the main axis, hyaline, smooth, usually bearing verticils of two to four branches arising from the stipe at an acute angle. Conidiophores more abundant on CMA than PDA. Aleurioconidia are pyriform to clavate or obovoid with a broad truncate basal scar, 2.8–4.6 × 1.7–3.2 μm (av = 3.7 × 2.5 μm, n = 50), in conidiophores separated by connective cells. Intercalary conidia are rare, pyriform to clavate, or subglobose, 2.7–4.6 × 1.9–2.7 μm (av = 3.5 × 2.3 μm, n = 7), in conidiophores separated by connective cells. Ascomata absent.

**Figure 6.**
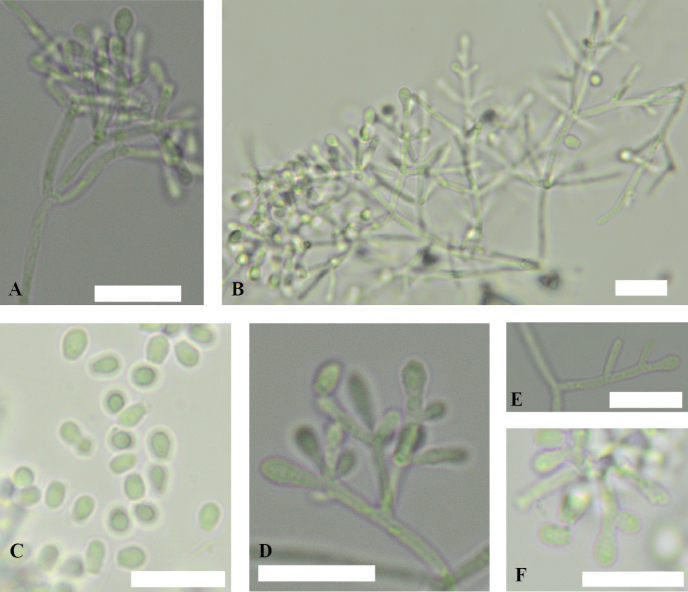
*Pseudogymnoascusramosus* sp. nov. **A, B, D** conidiophores **C** conidia **E, F** fertile hyphae bearing aleurioconidia. Scale bars: 10 µm.

##### Culture characteristics.

On OA, colonies reach 14 mm in diameter after 28 days at 15°C, round, slightly irregular, appressed, colorless to white, consisting of immersed and hyaline hyphae, small clumps of white cottony aerial mycelium, exudates and diffusible pigments absent; reverse white. On CMA, colonies reach 14 mm in diameter after 28 days at 15°C, round, slightly irregular, dense and slightly umbonate, floccose, white, abundant exudates in the form of transparent pale pink large droplets, diffusible pigments absent; reverse brown. On SDA, colonies reach 14 mm in diameter after 28 days at 15°C, round, slightly irregular, slightly raised and umbonate, floccose, shallow radial grooves, white at center, pink to white margin, exudates and diffusible pigments absent; reverse beige. On PDA, colonies reach 15 mm in diameter after 28 days at 15°C, irregular, raised, umbonate, floccose, white, dense, exudates initially in the form of transparent and colorless droplets and aging to dark red within two weeks, brown diffusible pigments; reverse brown. Growth occurred at 5°C and 15°C, with very minimal growth at 25°C; optimum growth was observed at 15°C. No culture attenuation was observed.

##### Distribution.

Mount Franke and Schroder Hill, Shackleton Glacier, Antarctica.

##### Ecology/substrate.

Cultured from Antarctic soil.

##### Additional specimen examined.

Schroder Hill, 508ASP, ibid.

##### Genbank accession numbers.

ITS = PQ453554, LSU = PQ453559, MCM7 = PQ497092, RPB2 = PQ497097, TEF1 = PQ497100.

##### NCBI BioSample Genome Accession.

SAMN40283454.

##### Note.

*Pseudogymnoascusramosus* has been placed as a member of clade Q (Figs 1, 2), which also includes unidentified species VKM F-4520 (Leushkin et al. 2015). Fig. 1 shows Q as sister to clade A, B, C, and D, however the placement of clade Q in relation to these clades has low bootstrap support. Leushkin et al. (2015) first introduced clade Q and had low bootstrap support for determining its overall placement with other clades in their gene tree. According to Fig. 2, there is strong bootstrap support for clade Q being sister to clades B, E, F, G, H, I, J, K; but data are missing for clades A, C, and D to confirm this placement. A unique characteristic of *P.ramosus* not documented in any described species of *Pseudogymnoascus* is its heavily branched hyphae with gregarious groupings of conidiophores. It is also relatively slow growing, only reaching 14 mm in diameter after 28 days on OA, CMA, SDA, and 15 mm on PDA. Phylogenetically, our *P.ramosus* isolates, strain 420ASP and 508ASP, form a well-supported independent lineage with a bootstrap value of 100, being the first described species in clade Q.

#### ﻿ Pseudogymnoascus russus

Taxon classificationAnimaliaThelebolalesPseudeurotiaceae

Childress & Quandt sp. nov

24F799FE-CDD5-56D7-8DB6-53B934CDE70A

856260

Figs 7 , 8

##### Etymology.

The name refers to the russet red color of exudates produced by colonies on PDA at 15°C.

##### Type.

Antarctica • Shackleton Glacier, Mount Wasko, 84°33'34.5"S, 176°48'40.38"W, 321 m, from soil, 4 Jan 2018, coll. I. Hogg, D. Wall & M. Diaz. Holotype 99ASP01, stored in a metabolically inactive state in the CFMR Herbarium, while ex-type metabolically active material is stored in the Reference Culture Collection at the CFMR.

**Figure 7.**
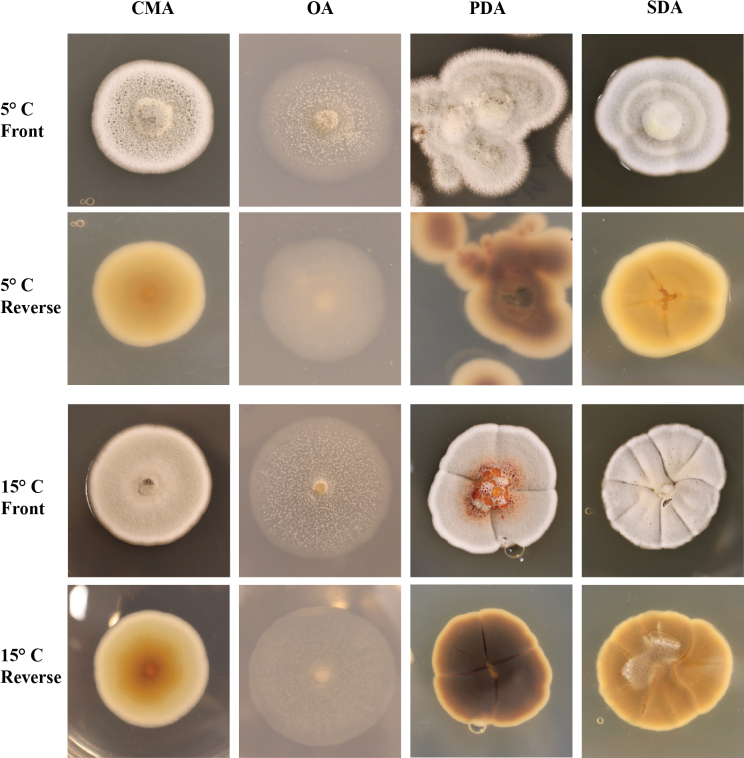
*Pseudogymnoascusrussus* sp. nov. colony morphology at 5°C and 15°C after 28 days on CMA, OA, PDA, and SDA.

##### Description.

On CMA and PDA hyphae branched, septate, hyaline, smooth, 0.9–2.1 μm wide. Racquet hyphae absent. Fertile hyphae bearing aleurioconidia, sessile or stalked, rarely bearing intercalary conidia. Arthroconidia not observed. Conidiophores abundant, solitary, generally erect, sometimes curved, arising in acute angles with the main axis, hyaline, smooth, usually bearing verticils of two to four branches arising from the stipe at an acute angle. Conidiophores more abundant on CMA than PDA. Aleurioconidia are pyriform to clavate or obovoid with a broad truncate basal scar, 2.8–4.8 × 2.1–3.4 μm (av = 3.7 × 2.8 μm, n = 50), in conidiophores separated by connective cells. Intercalary conidia are rare, pyriform to clavate, or subglobose, 3.0–4.6 × 2.1–3.2 μm (av = 3.76 × 2.5 μm, n = 13), in conidiophores separated by connective cells. Ascomata absent.

**Figure 8.**
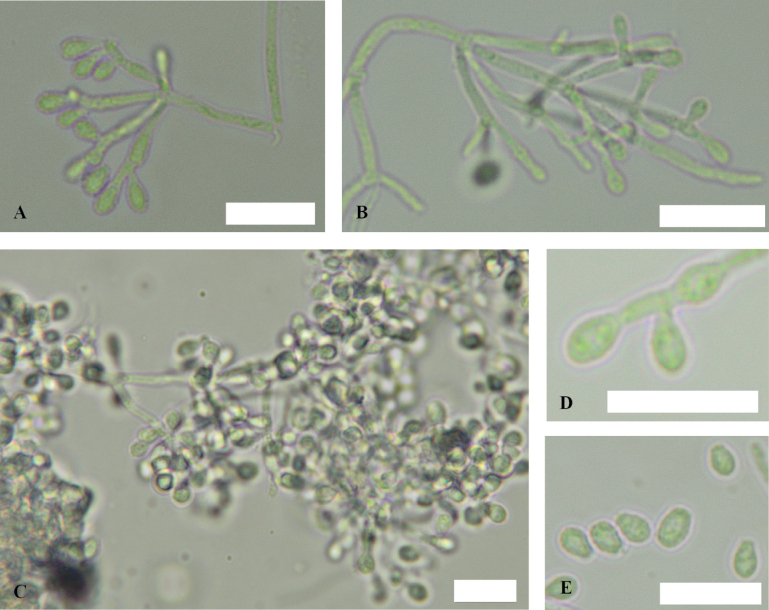
*Pseudogymnoascusrussus* sp. nov. **A, B, C** conidiophore **D** fertile hyphae bearing aleurioconidia **E** conidia. Scale bars: 10 µm.

##### Culture characteristics.

On OA, colonies reach 45 mm in diameter after 28 days at 15°C, round, appressed, colorless to white, consisting of immersed and hyaline hyphae, small clumps of white cottony aerial mycelium emerging throughout the colony, exudates and diffusible pigments absent; reverse white. On CMA, colonies reach 36 mm in diameter after 28 days at 15°C, round, flat, floccose, gray to white, margin filamentous and white, exudates and diffusible pigments absent; reverse rusty brown with white margin. On SDA, colonies reach 31 mm in diameter after 28 days at 15°C, irregular, raised, floccose, radial grooves, yellowish to gray, margin filamentous and white, exudates in the form of transparent and colorless droplets, diffusible pigments faint brown; reverse brown. On PDA, colonies reach 30 mm in diameter after 28 days at 15°C, irregular, slightly raised, umbonate, floccose, radial grooves, gray, margin filamentous and white, exudates in the form of large russet red droplets, diffusible pigments faint brown; reverse dark brown. Also on PDA, colonies reach 28 mm in diameter after 28 days at 25°C, slightly irregular/nearly round, raised, slightly umbonate, floccose aerial mycelium, light pink to gray to white, margin filamentous and white, exudates in the form of rust colored droplets, brown diffusible pigments; reverse dark brown. Conidial production was greater at 25°C compared to 15°C. Growth occurred at 5°C, 15°C, and 25°C; Growth rates were approximately equal at 15°C and 25°C. No culture attenuation was observed.

##### Distribution.

Mount Franke and Mount Wasko, Shackleton Glacier, Antarctica.

##### Ecology/substrate.

Cultured from Antarctic soil.

##### Additional specimen examined.

Shackleton Glacier, 390ASP, ibid.

##### Genbank accession numbers.

ITS = PQ453551, LSU = PQ453556, MCM7 = PQ497090, RPB2 = PQ497095, TEF1 = PQ497099,

##### NCBI BioSample genome accession.

SAMN40283452.

##### Note.

*Pseudogymnoascusrussus* has been placed as a member of clade B (Figs 1, 2). Clade B is comprised of *P.shaanxiensis*, *P.papyriferae*, *P.australis*, and *P.griseus* (Zhang et al. 2020, 2023b; Villanueva et al. 2021) Clade B also includes unidentified species RMFC101, 10KY12, 14PA06, 11MA08, 04NY17A, 24MN06, (Minnis and Lindner 2013) VKM F-4517 and VKM F-4515 (Leushkin et al. 2015). *Pseudogymnoascusrussus* can be differentiated from all other described species in clade B by its production of russet red colored exudate on PDA at 15°C. Additionally, it can be differentiated by *P.papyriferae*, *P.australis*, and *P.griseus* by its lack of arthroconidia. *Pseudogymnoascusrussus* can also be differentiated from *P.shaanxiensis* by its smaller hyphal width (0.9–2.1 μm vs. 1.5–2.5 μm). Phylogenetically our *P.russus* isolates, strains 99ASP01 and 390ASP, form a well-supported single clade with a bootstrap value of 88, separated from other taxa (Fig. 1). Sister to *P.russus* isolates there is an unidentified strain 11MA08; which we are not able to confirm whether it’s conspecific with *P.irelandiae* as we don’t have access to this isolate to analyze its culture and microscopic characteristics; however, we do know that their isolation sources are quite different, as 11MA08 was cultured from soil from bat hibernacula in Massachusetts, USA (Minnis and Lindner 2013).

## ﻿Discussion

In addition to the four species of *Pseudogymnoascus* described by Villanueva et al. (2021), *P.irelandiae*, *P.ramosus*, and *P.russus* are the only other novel species of Antarctic origin that have been described. Previously described species of Antarctic origin, cultured from marine sponges, are in clades B (*P.australis* and *P.griseus*), E (*P.lanuginosus*), and I (*P.antarcticus*), (Villanueva et al. 2021). Placement in both five gene (Fig. 1) and genome-scale (Fig. 2) phylogenetic analyses support *P.russus* as a member of clade B, *P.irelandiae* as a member of clade K, and *P.ramosus* as a member of clade Q, further providing evidence that there is not a single origin of *Pseudogymnoascus* in Antarctica. *Pseudogymnoascusramosus* is the first species to be described in clade Q, and *P.irelandiae* is the first to be described in clade K. Interestingly, *P.irelandiae* and strain A07MA10 represent the only clade within the genus *Pseudogymnoascus* with an amino acid insert in the same position in the TEF1 protein, first mentioned by Minnis and Lindner (2013), and shared with *Geomycesauratus* and *G.obovatus*.

While the members within clades agree between the *Pseudogymnoascus* five gene and protein phylogenies, five gene trees have historically lacked strong support for multiple internal nodes that determine the relationship between clades. Of particular relevance to this paper, clade K and clade Q lack this support (Fig. 1). Although there is strong support for the placement of clades K and Q in Fig. 2, multiple clades are missing genomic data to better resolve these placements. Full genomes from more *Pseudogymnoascus* species across clades A through Q are needed to generate stronger protein-based phylogenies that more accurately depict the relationships between clades proposed by Minnis and Lindner (2013) and Reynolds et al. (2016). Increased genomic sampling could also enable the identification of a more robust barcode gene, delimiting species, for the genus, as ITS alone is inadequate (Suppl. material 2).

While *Pseudogymnoascus* has gained considerable attention since the onset of white-nose syndrome with dozens of new species having been described (Zhang et al. 2020; Villanueva et al. 2021; Becker et al. 2023), there is a need for a more consistent and standardized way of describing novel species morphologies. Much of the literature has described novel species on PDA at 25°C after two weeks of growth (Zhang et al. 2020, 2021, 2023a, 2023b). However, species descriptions of *P.destructans*, *P.antarcticus*, *P.australis*, *P.griseus*, and *P.lanuginosus* found optimal growth temperatures to be below 15°C or 20°C (Gargas et al. 2009; Villanueva et al. 2021), thus categorizing these species as psychrophilic. Wang et al. (2017) studied strains of *P.destructans* and *P.pannorums.l.* collected from the Qinghai–Tibet Plateau with an optimal growth temperature of 4°C and 5°C respectively. *Pseudogymnoascusappendiculatus* and *P.verrucosus* have also been isolated from cold regions around the world including Antarctica (Rosa 2019), and were initially described by Rice and Currah (2006) growing at 15°C but the optimal growth temperature was not stated. While many fungal species cultured from Antarctica and other cold regions of the world are considered psychrophilic, some are psychrotolerant and may prefer warmer growth temperatures. For example, *P.russus* was isolated from Antarctic soils at 5°C but grew equally well at 15 and 25°C on PDA; Whereas *P.irelandiae* and *P.ramosus*, also isolated at 5°C, preferred a growth temperature of 15°C across all media types. Given that the growth optimum of *Pseudogymnoascus* species spans from 5°C to 25°C, it is recommended that future studies determine and report the growth optimum of each newly described species and describe the culture characteristics at that temperature.

**Figure 9.**
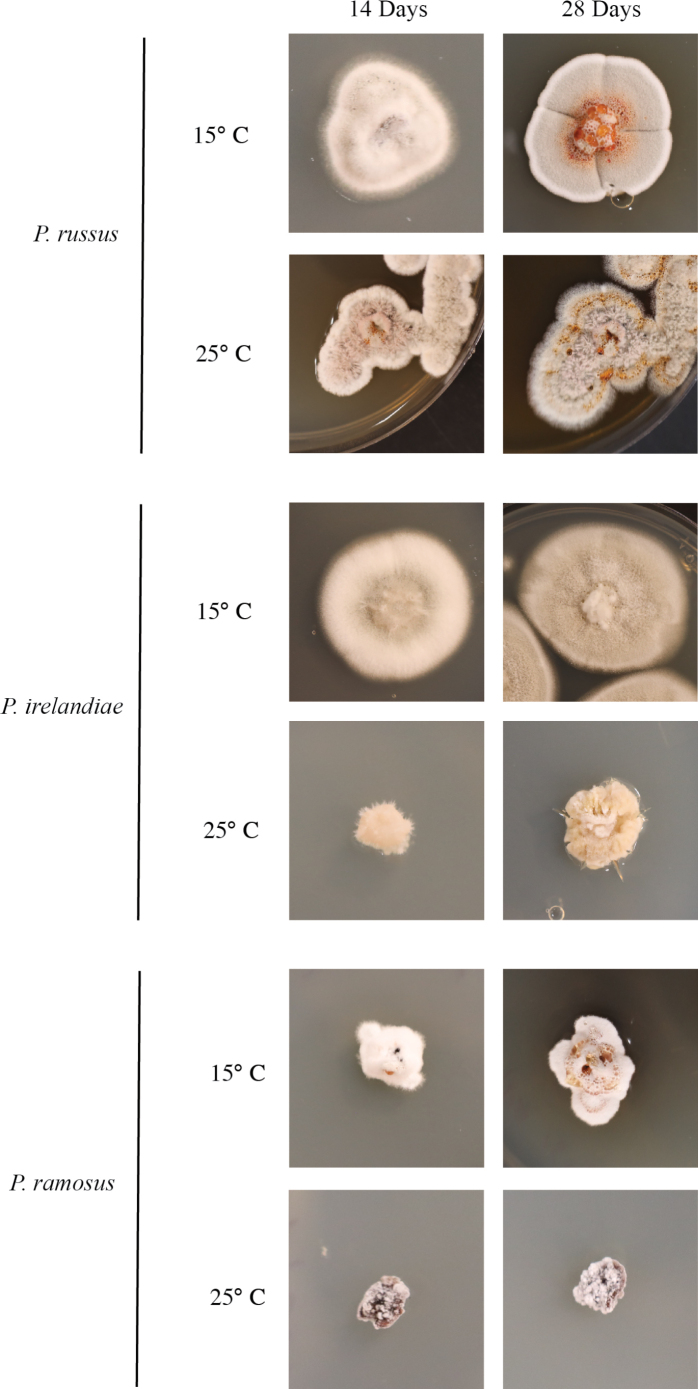
*P.russus*, *P.irelandiae*, and *P.ramosus* growing on PDA at 15°C and 25°C at 14 days and 28 days.

*Pseudogymnoascus* colony morphology necessary for describing novel species varies greatly depending on temperature, media type, and age (Fig. 9). While *P.russus* grew to similar colony diameters at 15°C and 25°C on PDA, colony morphologies differed. At 25°C, after both two and four weeks of growth, colonies were more floccose, pink in color, had aerial mycelium, had more conidial production overall, had darker rust colored exudate rather than russet red, and had diffusible brown pigmentation. After two weeks of growth at 15°C on PDA, very small clear exudates were present and became much larger and russet red colored after four weeks of growth, the colony also became more gray in color with time. At 25°C on PDA the amount of exudate increased but remained rust colored. As *P.irelandiae* and *P.ramosus* are psychrophilic with optimal growth observed at 15°C, very little growth occurred at 25°C over time and important colony characteristics were not observable. *Pseudogymnoascusirelandiae* and *P.ramosus* began producing small droplets of exudate at two weeks and became larger and more easily observable after four weeks. As shown in Fig. 9, it is recommended that novel *Pseudogymnoascus* be described after enough time has elapsed, often four weeks, to best observe colony morphology.

## ﻿Conclusions and future directions

This study expands our understanding of the genus *Pseudogymnoascus* by describing three novel species, *P.russus*, *P.irelandiae*, and *P.ramosus*, isolated from the unique and extreme environments of Antarctic soils. These findings not only contribute to the growing catalog of *Pseudogymnoascus* diversity but also highlight the genus’ remarkable presence in harsh conditions and frequent preference for cold growth conditions. This study also suggests that a variety of cold environments, in addition to bat hibernacula where *Pseudogymnoascus* is most commonly studied, are likely to harbor undescribed *Pseudogymnoascus* diversity. To better understand the diversity of this genus, more research across a variety of cold habitats throughout the world is necessary.

Many *Pseudogymnoascus* strains cultured from Antarctica remain undescribed (Rosa 2019). More research is necessary to not only understand the origins of *Pseudogymnoascus* in Antarctica, but also elucidate their biological activity and their ecological roles in Antarctic soils. Future work studying the genomes generated as a part of this study should examine genes involved in psychrotolerance and other adaptations of polar fungi. Additionally, the generation of additional *Pseudogymnoascus* genomes across clades A through Q will help to resolve the evolutionary history of this genus and relationships between the clades. Our work demonstrates that it is advantageous to describe novel *Pseudogymnoascus* species at their optimal growth temperature and to observe growth for at least 28 days. Growth on PDA at a minimum is recommended so that novel species can be compared to the majority of previously described species. Growth on OA is generally important to observe the presence or absence of ascomata and, for species described in this paper, conidia were produced most abundantly on CMA. The description of Antarctic fungi is a foundational step for additional research to reveal further unknown biodiversity and to offer clues about microbial survival, adaptations, and ecological roles in extreme environments.

## Supplementary Material

XML Treatment for Pseudogymnoascus irelandiae

XML Treatment for Pseudogymnoascus ramosus

XML Treatment for Pseudogymnoascus russus

## ﻿Additional information

### Adherence to national and international regulations

All the fungal strains used in this study have been legally obtained, respecting the Convention on Biological Diversity (Rio Convention).

### Author ORCIDs

Mary K. Childress https://orcid.org/0009-0004-4625-3617

Nicholas B. Dragone https://orcid.org/0000-0002-8983-6482

Benjamin D. Young https://orcid.org/0000-0002-5195-9399

Byron J. Adams https://orcid.org/0000-0002-7815-3352

Noah Fierer https://orcid.org/0000-0002-6432-4261

C. Alisha Quandt https://orcid.org/0000-0003-0260-8995

### Data availability

All gene sequences generated in this study can be found in Genbank. The accession numbers of the sequences deposited in Genbank are ITS: PQ453551–PQ453555, LSU: PQ453556–PQ453560, MCM7: PQ497089–PQ497093, RPB2: PQ497094–PQ497098, and TEF1: PQ497099–PQ497101. All raw reads and assembled genomes generated in this study are available on the NCBI at project number PRJNA1084838. All generated genomes, predicted proteins, and annotations are also available at https://doi.org/10.5281/zenodo.14037014. All aligned gene fasta files can be found at https://zenodo.org/records/14834766.

## Supplementary materials

Supplementary material 1 Suppl. tables S1–S7 Data type xlsx Explanation note **table S1**: Collection details; **table S2**: Primer sequences; **table S3**: PCR Settings; **table S4**: Five gene phylogeny genbank accession numbers; **table S5**: Protein phylogeny NCBI genome accesion numbers; **table S6**: Morphology of *Pseudogymnoascus* Isolated from Antarctica; **table S7**: Genome statistics. https://binary.pensoft.net/file/1287502This dataset is made available under the Open Database License (http://opendatacommons.org/licenses/odbl/1.0/). The Open Database License (ODbL) is a license agreement intended to allow users to freely share, modify, and use this Dataset while maintaining this same freedom for others, provided that the original source and author(s) are credited.Mary K. Childress, Nicholas B. Dragone, Benjamin D. Young, Byron J. Adams, Noah Fierer, C. Alisha Quandt

Supplementary material 2 Maximum likelihood (ML) ITS phylogeny of *Pseudogymnoascus* Data type pdf https://binary.pensoft.net/file/1287503This dataset is made available under the Open Database License (http://opendatacommons.org/licenses/odbl/1.0/). The Open Database License (ODbL) is a license agreement intended to allow users to freely share, modify, and use this Dataset while maintaining this same freedom for others, provided that the original source and author(s) are credited.Mary K. Childress, Nicholas B. Dragone, Benjamin D. Young, Byron J. Adams, Noah Fierer, C. Alisha Quandt

Supplementary material 3 Maximum likelihood (ML) ITS phylogeny of all *Pseudogymnoascus* isolates obtained in this study Data type pdf https://binary.pensoft.net/file/1287504This dataset is made available under the Open Database License (http://opendatacommons.org/licenses/odbl/1.0/). The Open Database License (ODbL) is a license agreement intended to allow users to freely share, modify, and use this Dataset while maintaining this same freedom for others, provided that the original source and author(s) are credited.Mary K. Childress, Nicholas B. Dragone, Benjamin D. Young, Byron J. Adams, Noah Fierer, C. Alisha Quandt

Supplementary material 4 Maximum likelihood (ML) phylogeny of *Pseudogymnoascus* based on a concatenated dataset of four genes (ITS, LSU, MCM7, RPB2) Data type pdf https://binary.pensoft.net/file/1287507This dataset is made available under the Open Database License (http://opendatacommons.org/licenses/odbl/1.0/). The Open Database License (ODbL) is a license agreement intended to allow users to freely share, modify, and use this Dataset while maintaining this same freedom for others, provided that the original source and author(s) are credited.Mary K. Childress, Nicholas B. Dragone, Benjamin D. Young, Byron J. Adams, Noah Fierer, C. Alisha Quandt
