# Supplementary figures and images for: ﻿Three new Pseudogymnoascus species (Pseudeurotiaceae, Thelebolales) described from Antarctic soils
Source: IMA Fungus. 2025 Mar 21;16:e142219. doi: 10.3897/imafungus.16.e142219 (PMC11953729; doi:10.3897/imafungus.16.e142219)

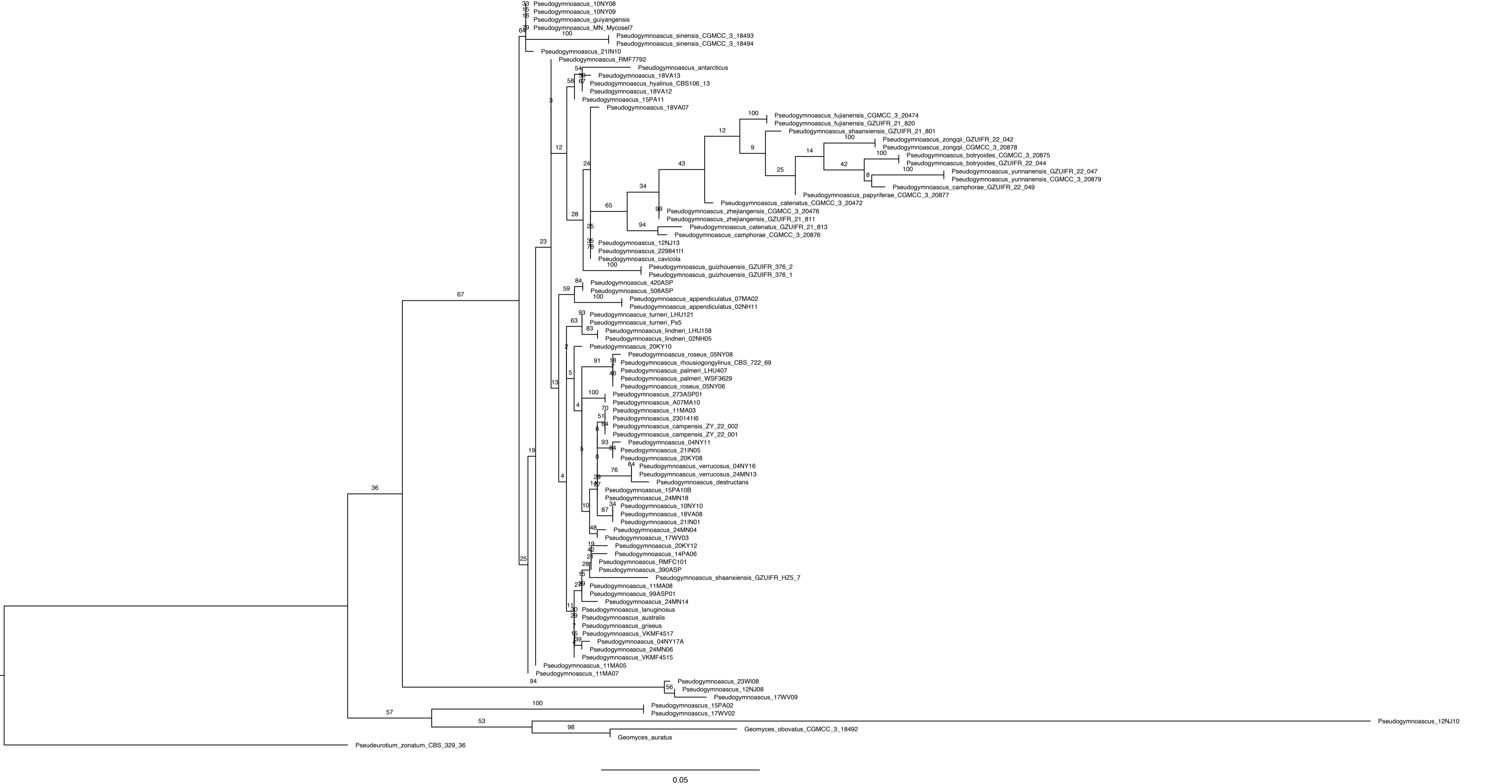

Supplementary Figure 1. Maximum likelihood (ML) ITS phylogeny of *Pseudogymnoascus*.

Supplement: Supplementary material 2 — Maximum likelihood (ML) ITS phylogeny of Pseudogymnoascus [file imafungus-16-e142219-s002.pdf]
