# Supplementary material for: ﻿Three new Pseudogymnoascus species (Pseudeurotiaceae, Thelebolales) described from Antarctic soils
Source: IMA Fungus. 2025 Mar 21;16:e142219. doi: 10.3897/imafungus.16.e142219 (PMC11953729; doi:10.3897/imafungus.16.e142219)
Supplement: Supplementary material 3 — Maximum likelihood (ML) ITS phylogeny of all Pseudogymnoascus isolates obtained in this study [file imafungus-16-e142219-s003.pdf]

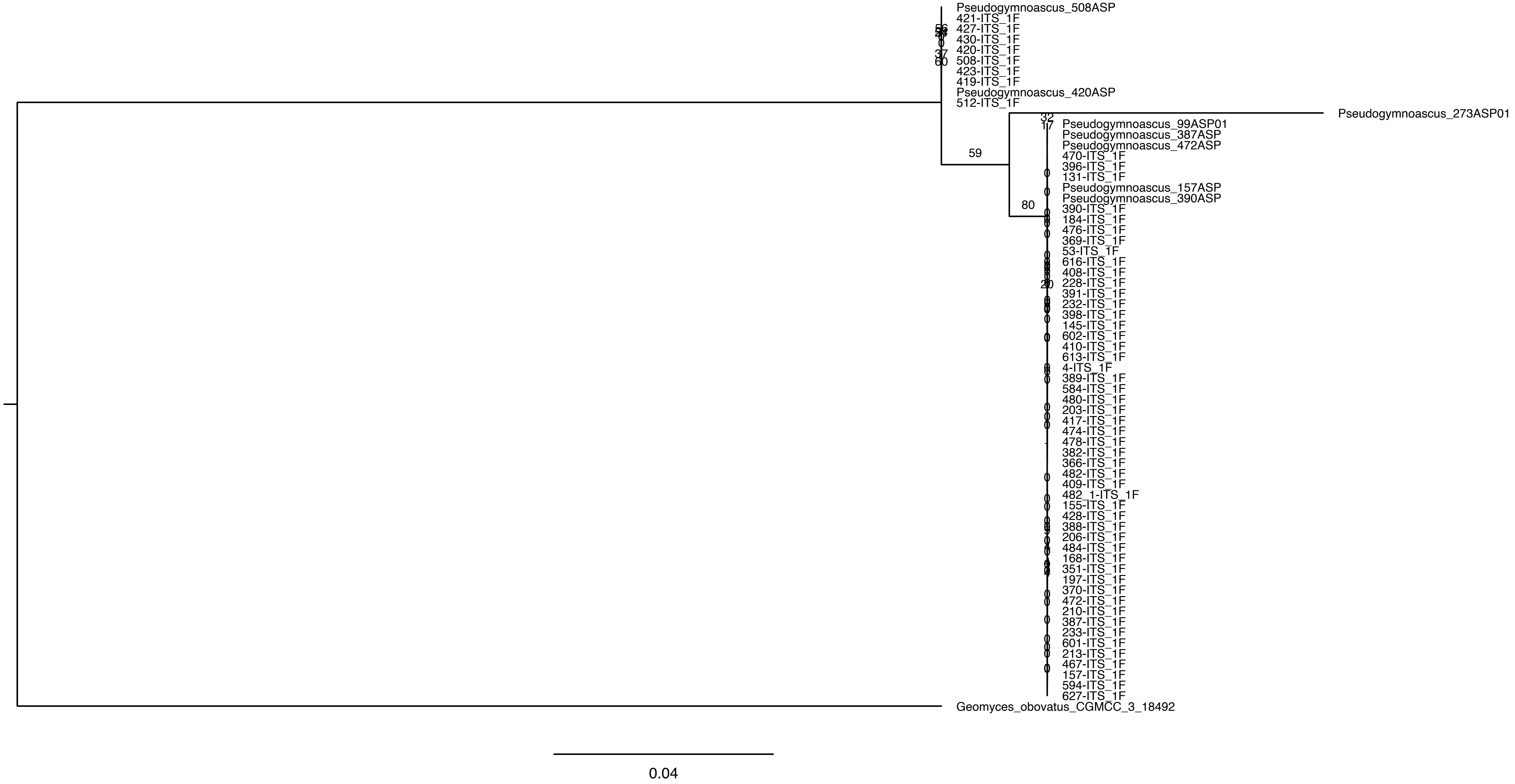

Supplementary Figure 2. Maximum likelihood (ML) ITS phylogeny of all *Pseudogymnoascus* isolates obtained in this study.
