# Supplementary material for: ﻿Three new Pseudogymnoascus species (Pseudeurotiaceae, Thelebolales) described from Antarctic soils
Source: IMA Fungus. 2025 Mar 21;16:e142219. doi: 10.3897/imafungus.16.e142219 (PMC11953729; doi:10.3897/imafungus.16.e142219)
Supplement: Supplementary material 4 — Maximum likelihood (ML) phylogeny of Pseudogymnoascus based on a concatenated dataset of four genes (ITS, LSU, MCM7, RPB2) [file imafungus-16-e142219-s004.pdf]

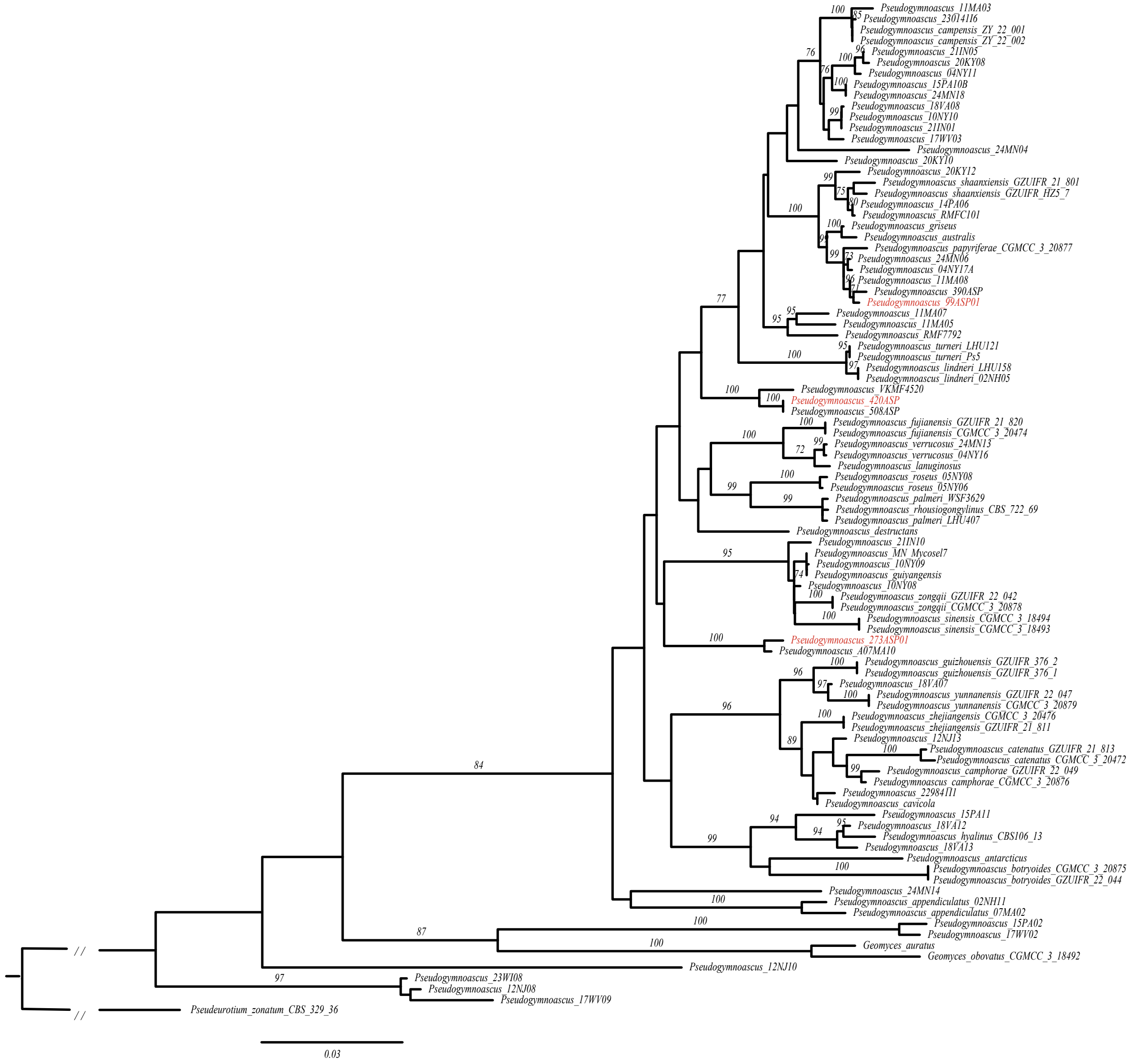

Supplementary Figure 3. Maximum likelihood (ML) phylogeny of *Pseudogymnoascus* based on a concatenated dataset of four genes (ITS, LSU, MCM7, RPB2). New species described here are highlighted in red.
